# The “Visual Shock” of Francis Bacon: an essay in neuroesthetics

**DOI:** 10.3389/fnhum.2013.00850

**Published:** 2013-12-10

**Authors:** Semir Zeki, Tomohiro Ishizu

**Affiliations:** Wellcome Laboratory of Neurobiology, Cell and Developmental Biology, University College LondonLondon, UK

**Keywords:** face perception, body perception, Francis Bacon, neuroesthetics, face and body deformation

## Abstract

In this paper we discuss the work of Francis Bacon in the context of his declared aim of giving a “visual shock.”We explore what this means in terms of brain activity and what insights into the brain's visual perceptive system his work gives. We do so especially with reference to the representation of faces and bodies in the human visual brain. We discuss the evidence that shows that both these categories of stimuli have a very privileged status in visual perception, compared to the perception of other stimuli, including man-made artifacts such as houses, chairs, and cars. We show that viewing stimuli that depart significantly from a normal representation of faces and bodies entails a significant difference in the pattern of brain activation. We argue that Bacon succeeded in delivering his “visual shock” because he subverted the normal neural representation of faces and bodies, without at the same time subverting the representation of man-made artifacts.

## Introduction

Neuroesthetics seeks inspiration and insight from works of art and from debates in the humanities to try to gain some insights, however small, into the workings of the brain. The present article, on the work of the British painter Francis Bacon, is written in the pursuit of that aim. The article does not delve into the artistic merits of Bacon's works, which lies more in the province of art criticism; it does not discuss the artistic influences that shaped Bacon's art, which belongs more properly to art history; nor does it consider, except in a marginal sense, the influence of Bacon's up-bringing and sexual orientation on his art, which would trespass into psycho-analytic studies. Instead, concentrating above all on his artistic output as well as on statements about his work from him and others, we try to ask how what his declared aim, of trying to give “a visual shock,” amounts to in neural terms and what insights into brain organization the resultant work gives.

## A visual shock

Bacon, whose first US exhibition was described in *Time* (October 19, 1953) as a “chamber of horrors” filled with paintings that are “snapshots from hell,” told Melvyn Bragg (1985) on the *South Bank Show* that he wanted to give a “shock… not a shock that you could get from the story [but] a visual shock.” He apparently succeeded in doing so, not only when he first began to produce his work but even today. In the late 1940s, when he first began to exhibit, a critic wrote in *The Observer* that Bacon's paintings “… horrifying though they” are also technically superb, making one “… regret the more that the artist should have been brought to subjects so esoteric” (quoted in Peppiatt, 1996, p 156), while the correspondent of *The Times* thought the subject of his pictures to be “so extremely repellent” as to make his paintings “as vivid and as meaningless as a nightmare,” lamenting that Bacon should have used his considerable powers of imagination and pictorial skill to produce something “which it is impossible not to think worse than nonsense, as *Head II*, which appears to be a mutilated corpse, most certainly is” (Peppiatt, 1996, p 156). Nor are such comments restricted to the early phase of Bacon's output; they persist until the 1990s, well after he had acquired world-wide fame. This suggests that the passage of time did not diminish the intensity of the visual shock that he intended to produce, either in the average viewer or among those more knowledgeable about art. The reaction of the average viewer is perhaps best summed up by Margaret Thatcher (1992), who described him as “that man who paints those dreadful pictures.” This view is not too distant from those expressed in even more powerful adjectives by more learned critics, Margaret Walters (Cork, 1985) describing his work as, “daemonic, hysterical, monstrous” and Peter Fuller describing him as an “evil genius” whose images were “odious” (Brighton, 2001). As recently as 2012 he was described in *The Guardian* as creating “a monstrous, surreal imaginative world of enclosed rooms and private hells” (Jones, 2012). Such adjectives leave little doubt that he had succeeded in producing an enduring shock, even in the same viewer.

The conceptual framework within which Bacon worked is relatively easy to establish and of importance to our argument. It is significant that, like many other great artists, he destroyed many of his paintings, claiming that he had usually destroyed the better ones (Sylvester, 1963). He was always trying, he said, to paint the one perfect image which, he claimed, he had never succeeded in achieving. Thus, by his own account, all these paintings were a journey toward the representation, in a single perfect image that was never achieved, of a concept in his mind. He claimed to have had a concept in mind before starting work on a painting but that, once he started, the painting changed unpredictably and by accidents, but accidents “out of which [the artist] chooses the marks which he wants to leave” (Jebb, 1965) (that is, those marks that correspond best to his concept), which for him were “forms that relate to the human image but are a complete distortion of it” for only then could one get “to the reality behind the image” (Sylvester, 1963). From those “accidents” he thus chose what came closest to representing his concept.

### Bacon's overall concept

What was the overall concept in his mind? It is useful to begin by making a distinction between inherited and acquired brain concepts (Zeki, 2008). One of the primordial functions of the brain is to acquire knowledge, and it does so through inherited and acquired concepts. Faces and bodies are examples of the former and there is reasonable evidence to suggest that the recognition of faces and bodies, though not of their identity, is at least facilitated through inherited concepts that are present at birth (Zeki, 2008) (see section The Privileged Status of Faces and Bodies in Visual Perception). Inherited concepts are robust, stable and do not change with time or do so insignificantly; crucially, they are common to all humans, except in relatively rare pathological conditions, of which acquired prosopagnosia is especially noteworthy in this context (see section Prosopagnosia or Facial Imperception). Certain configurations and relationships are critical for recognition of faces and bodies as normal ones. By contrast, acquired concepts to which that of houses, cars and other human artifacts and situations belong, are malleable and change with time and acquired experience and are culture dependent. At any given moment, therefore, they are the synthesis of all previous experiences of the same category of object or situation. (Zeki, 2008).

Bacon said that he tried to represent “concentrations of reality” (Bragg, 1985). We may surmise from his work that one such “concentration of reality” (which we equate with acquired concepts) behind the images that he produced was that of alienation, a situation in which he commonly found himself and apparently saw in others. The sense of alienation may have been the result of his own tastes which, during much of his lifetime, were regarded by Church, state and society as an evil which should carry a deep sense of guilt. According to Andrew Brighton (2001), Bacon found inspiration in the writings of Count Joseph de Maistre, an 18th century French philosopher who had emphasized universal guilt derived from Original Sin and the Fall. Thus, the lonely, alienated, figures in Bacon's paintings (and most of his paintings contain single figures, some two, rarely more) were part of mankind, bearing a guilt common to all even if differing in detail and traceable to different sources, allowing Bacon to believe that he was depicting a universal message, that of pain. For Bacon, “nearly all reality is pain” and he thought that, when we look at his paintings, we are looking at the real world: “What could I make,” he asked, “to compete with what goes on every single day… except that I may have tried to make images of it; I have tried to re-create it and make, not the horror, but… images of realism” (Bragg, 1985).

The means that Bacon employed to project his acquired concept in his paintings was to subvert the brain's inherited concepts of what bodies and faces should look like. Thus, in addition to the lonely figures, he made use of mutilated and savaged faces and bodies, often in combination. This enabled him, in his own words, to hit “the nervous system more violently and poignantly” and thus get to the reality behind the image (Sylvester, 1963). He was looking, it seems, for something primitive and instantaneous, divorced as much as possible from the cognitive element and presumably from cultural context as well, for by concentrating on deformed faces and bodies he was working outside any social and cultural context and within one that most, irrespective of race or culture, would respond to, even if only negatively. Faces and bodies occupy a very privileged position in visual perception, and indeed their recognition may be due to inherited brain concepts. Objects do not share that same privileged position and hence their distortions would not produce the same visual shock or, if they do, they become rapidly adapted to, unlike distorted faces and bodies (Chen and Zeki, 2011). Bacon, on whom Picasso was a leading influence, thus violated and subverted deliberately the brain template for registering faces and bodies, leading to an almost universal experience of his portraits and bodies as disturbing. By contrast, Picasso's Cubist work is not as disturbing, partly because many of his portraits do not disfigure or mutiliate faces or distort the relationship between their components as violently as Bacon; disfigurations are minimal and maintain significant parts of the relationships between components intact, even when presenting, or attempting to present, different views on the same canvas. The adjectives describing Bacon's work, which are peppered throughout this article, testify that few, if any, have qualified these works as beautiful, even if they consider them to have considerable artistic merit; almost all find them disturbing. These disfigured and mutilated faces and bodies are usually set against neutral backgrounds or anonymous spaces containing few objects—chairs, tables, light bulbs, cars—which, by contrast, are not in any way deformed. He seems to have had a marked preference for faces even in other artists' work; for example, he preferred the portraits of both Picasso and Giacometti to their other work (Archimbaud, 1992).

That Bacon should have concentrated almost exclusively on distorted human bodies and faces to produce an immediate emotional impact on the nervous system, before things got “spelled out” in the brain (Peppiatt, 1996), invites enquiry into what is so special about the neural representation of faces and bodies, which they do not share with other everyday objects. One question we therefore address is whether there is any neurological basis for this violent, primitive and instantaneous assault, an assault that lies beyond reasoning. It was always Bacon's intent not to appeal to reason or even to thinking. The paintings, stripped of any associations, contained the message and his concept, but otherwise had no story to tell for, as he said, “once an image could be explained… it was worthless,” adding that, “After all, if you could explain it, why would you go to the trouble of painting it” (Peppiatt, 1996, p. 117); in his paintings, he was presenting, he said, “nothing except what people wanted to read into it” (Bragg, 1985). The central argument in this essay, which we develop below, is therefore that Bacon was trying, in his work, to project his acquired concept of pain and alienation and horror by subverting, as far as is possible, the brain's inherited concepts of face and body; that, in other words, he was trying to use an inherited brain concept to project his own acquired concept.

To achieve his overall concept in paintings, that of depicting realism by subverting the brain's inherited concepts, Bacon worked from memory and from photographs but frequented establishments such as the Colony Club in London, where people, as he told Melvyn Bragg (1985), were completely dis-inhibited and not on their guard, so that he could study them in the raw, as it were. As well, he was fascinated with movement, especially as portrayed in Edweard Muybridge's chronophotography of the movement of deformed animals as well as in the “Extraordinary photographs of animals taken out just before they were slaughtered” (Sylvester, 1963). This obsession with deformity and violence extended to his literary tastes. One of his favorite literary sources was the *Oresteia* by Aeschylus. It was, he said, “the most blood-bathed tragedy that exists, with almost nothing but blood from beginning to end” and yet, “The reek of human blood smiles out at me” was a favorite passage of his from the play (Peppiatt, 1996, p 111). The preoccupation with deformity, violence and violent distortions, indeed with representing violence (for almost all his paintings suggest that a violence has been done to the subject) may have been the result of several factors: the violence he received from his father, to whom he was sexually attracted, the “neurosis” of the century in which he lived and his experiences as an orderly during the Second World War, his own taste for violence even in sex, which he considered to be a violent act. Whatever the cause, he was partial to portraying the human condition by representing violence, for he considered the whole of life—from birth to death—to be violent.

We first address the question of whether faces and bodies occupy a privileged position in visual perception because of inherited brain concepts regulating their recognition, one not shared by objects and, next, whether distortion of faces and bodies influences the neural response more than distortion of objects and man-made artifacts. The relevance of discussing this in the context of this article is our belief that inherited brain concepts, such as configurations that qualify a stimulus as a face or body, are much more susceptible to the effects of distortion than acquired ones, to which houses, cars and man-made objects in general belong (Zeki, 2008; Chen and Zeki, 2011), and that Bacon consistently achieved his effects by distorting inherited brain concepts of face and body and sparing the objects, which are more resistant to distortion.

## Faces and bodies

Faces in general occupy a very privileged position in visual perception, as do bodies. This is not surprising, given their importance in obtaining knowledge about an individual, their emotional status at any given moment and their identity. The literature on the topic of face perception is now quite voluminous, and the one on body perception tending in that direction. We do not provide an exhaustive review here but distil from it those points that are especially relevant for discussing Bacon's “visual shock” and its enduring effect, in terms of that privileged position.

### The privileged status of faces and bodies in visual perception

**Figure 1:**
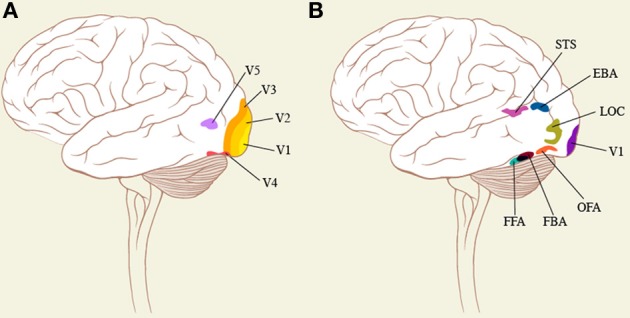
**(A)** shows some of the classical visual areas on a surface drawing of the brain, while **(B)** shows the areas that are critical for face and body recognition. The position of these areas is approximate.

Reflecting their significance for acquiring knowledge, special areas of the brain appear to be critical for the recognition of faces and bodies, although whether these areas are uniquely specialized for faces or bodies has been debated (Haxby et al., 2001) as has the question of whether there is an inherited neural template for facial recognition, some considering that it is more a matter of expertise derived from intimate contact and experience (Gauthier and Nelson, 2001; Bilalic et al., 2011). Whichever view turns out to be correct, there is common agreement that the areas enumerated below are strongly activated by faces. Among these are (i) an area located in the fusiform gyrus and known as the fusiform face area (FFA) (Sergent et al., 1992; Kanwisher et al., 1997; Kanwisher and Yovel, 2006) (Figure 1B), damage to which leads to the syndrome of *prosopagnosia* or an incapacity to recognize familiar faces (Damasio et al., 1982, for a review). We note in passing that the FFA is also activated by faces viewed from different angles (e.g., Pourtois et al., 2005) and by animal faces (Maguire et al., 2001), both common in Bacon's work. (ii) an area located in the inferior lateral occipital gyrus and known as the occipital face area (OFA) (Peelen and Downing, 2007; Pitcher et al., 2011) and (iii) a third area, located in the superior temporal sulcus, which appears to be involved in the recognition of changing facial features and expressions (Haxby et al., 2000; Kanwisher and Yovel, 2006), thus emphasizing the importance of the face as a means of obtaining knowledge about a person's emotional status. These areas respond better to faces and give weaker or no responses when the faces are scrambled so as to contain all the elements but arranged in a way that is different and does not lead to recognition of a face (Kanwisher et al., 1997). This in itself, at a very elementary level, implies that there must be certain configurations of a stimulus if it is to lead to activity in areas critical for the recognition of faces. The privileged status of face perception is further emphasized by the very rapid activation of OFA, at 60–100 ms after stimulus onset (Pitcher et al., 2007).

**Figure 2:**
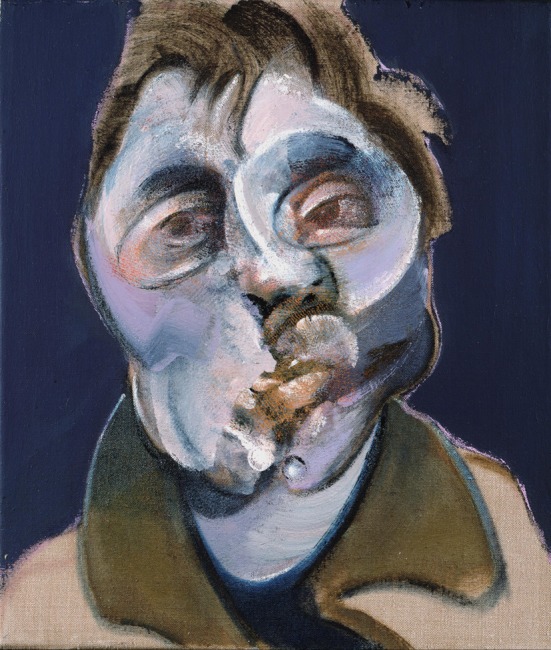
**Francis Bacon—*Self Portrait*, 1969, an example of a mis-aligned face**. © The Estate of Francis Bacon. All rights reserved. DACS 2013.

That there is a privileged mechanism that favors the early recognition of faces and bodies is further supported by evidence which shows that the face and body recognition systems are not only very robust but also very exigent in their demands for activation. For example, the negative EEG potential at 170 ms (which refers to a negative deflection, N170, of occipito-temporal origin, occurring at about 170 ms after presentation of the stimulus, and is larger in amplitude to faces and bodies than to objects) is demanding as to the correct configuration of the face since mis-aligning the two halves of a face delays and increases it specifically for upright faces, much less so for inverted ones (Ishizu et al., 2008). Here it is interesting to note that many, if not most, of Bacon's portraits can arguably be said to be misaligned in one way or another (see Figure 2). One may surmise from this that a stimulus such as that of Figure 2 would equally delay and increase the 170 ms deflection, in other words signal an abnormal configuration by leading to a modified pattern of neural responses.

The N170 component is also enhanced and delayed when the stimuli are those of inverted bodies (Stekelenburg and de Gelder, 2004; Minnebusch et al., 2008), thus suggesting an interaction between separate representation of faces and bodies, since images of human bodies themselves elicit a negative peak at 190 ms which differs in spatial distribution (Thierry et al., 2006; Ishizu et al., 2010); how a mutilated head sitting on a mutilated body, as is common in Bacon's work, would affect neural responses is not known, the effects of distortion having been studied in relation to a face or a body but not the two together. All of this speaks in favor of an essential configuration for faces, which may be due to an inherited or rapidly acquired template for facial recognition.

That even severe distortion of faces (and bodies) such as Bacon regularly practiced has little effect, beyond a delay, on the recognition of a stimulus as a face or a body testifies to the robustness of the representation, even if distorted faces result in a pattern of activity in the brain that is different from that obtained with neutral faces (see section A Fast Route for the Recognition of Facial and Body Stimuli). Hence the face recognition system is robust on the one hand and susceptible to disfiguration on the other, since disfiguration leads to a different pattern of neuronal activity.

**Figure 3:**
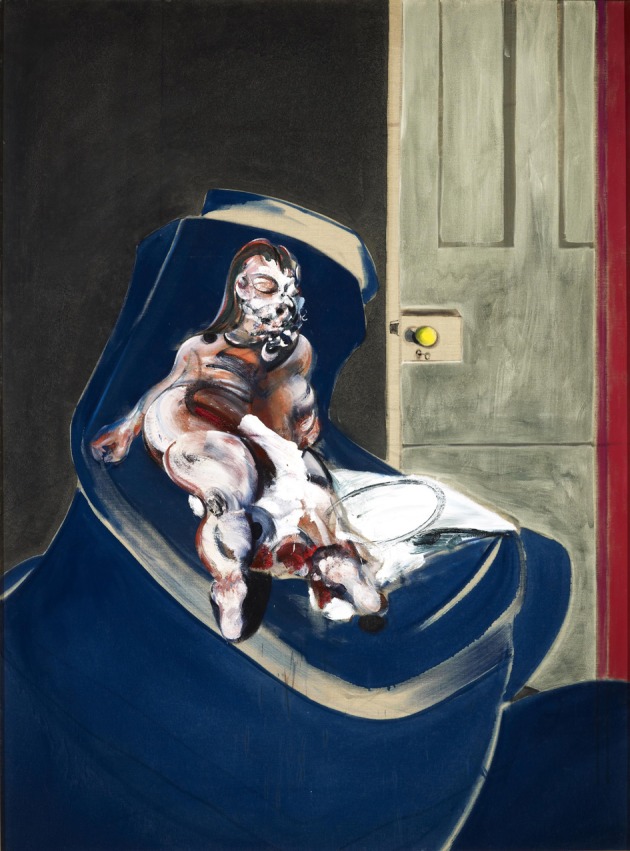
**Francis Bacon—*Portrait of Henrietta Moraes on a Blue Couch*, 1965, an example of disfigured face and body**. © The Estate of Francis Bacon. All rights reserved. DACS 2013.

The brain also appears to devote special cortical areas to the representation of human bodies, even headless ones (Schwarzlose et al., 2005). One of these is the fusiform body area (FBA), located in the fusiform gyrus in close proximity to the FFA, and the other is the extrastriate body area (EBA) located in the infero-posterior part of the temporal cortex, neighboring area OFA (Peelen and Downing, 2007 for a review) (see Figure 1B). Hence, there is also an essential configuration that is critical for eliciting activity from these specialized areas. But here again, Bacon, though maintaining the relationship between the constituents that constitute a body, distorted them severely and added a subversive emotional envelope (see section The Effect of Distortions of Face and Body on Cortical Activity). The areas critical for body recognition lie in close proximity to those for facial recognition (the OFA and the FFA); the brain thus appears to devote separate systems to the recognition of bodies and of faces but ones that are intimately connected since exposure of subjects to pictures of fearful body expressions activates the FFA (Hadjikhani and de Gelder, 2003), implying an intimate anatomical and functional connection between them. We note in passing that, his portraits apart, Bacon commonly disfigured both faces and bodies in single compositions (see Figure 3).

The areas enumerated here may not be the only ones that are important in the recognition of faces and bodies, and their emotional status; some have argued that the recognition of faces engages a much more distributed system (Ishai et al., 2005), but there is common agreement that they are critically important. Hence, viewing of Bacon's portraits is strongly dependent upon the functioning of these areas, an interesting if by now obvious fact. It has, however, also been argued that, even within the region of the fusiform gyrus occupied by the FFA, cells responsive to common objects may be found (Haxby et al., 2001). This is interesting, both in the context of Bacon's work and in relation to the neurobiology of visual representation in the brain. Given the resistance of objects, and the susceptibility of faces and bodies, to inversion and to distortion (see below), it becomes interesting to enquire whether cells representing faces and bodies on the one hand and objects on the other, are regulated differently, even if they co-occur in the same area(s) and whether it is because of this differential susceptibility that Bacon concentrated on deforming faces and bodies and sparing objects.

## Prosopagnosia or facial imperception

Prosopagnosia or an incapacity to recognize an individual through the face, and especially inherited prosopagnosia (McConachie, 1976; Ariel and Sadeh, 1996), also supports the view that there is an inherited or a rapidly acquired template for face representation that is not shared by objects. When acquired, the syndrome is usually the result of damage to the fusiform gyrus that includes the FFA. Prosopagnosia may result in an incapacity limited to the recognition of familiar faces but there have been examples of patients simply not able to recognize faces. The imperceptions may extend to an inability, or impaired ability, to recognize the faces of animals (Assal et al., 1984), which have a basic significant facial configuration not unlike humans, and we note here that Bacon depicted both human and animal faces and bodies, sometimes in combination. Not even knowledge that a prosopagnosic patient is actually looking at a face (for example at his own in a mirror) can restore the normal perception of a face (Pallis, 1955).

For our purposes here, we may summarize this section by saying that, regardless of disagreements over important details, there is now general agreement that the face and body recognition systems are neurologically robust and that several cortical areas are critical for their recognition. The relevance of a robust system is that its properties are much less plastic and therefore much less modifiable with experience, a point that seems to us of importance in understanding how Bacon was able to produce a visual shock.

### Form representation in the brain

The form system in the brain is commonly thought to be derived from the orientation selective cells of V1 (Hubel and Wiesel, 1977) (Figure 1A) and consists of a single hierarchical pathway which uses the orientation selective cells to build up more complex forms, and eventually complex objects that an area such as the lateral occipital complex (LOC) responds to (Grill-Spector et al., 2001). This view is almost certainly far too simplistic and there is evidence that the form system itself may consist of parallel sub-systems. We do not review this here but point to clinical evidence which shows that (a) agnosias for complex shapes and objects need not be accompanied by an agnosia for simple line representation of the same shapes (Humphreys and Riddoch, 1987) and, conversely, that agnosia for simple line drawings of complex shapes need not be accompanied by an agnosia for the complex shapes themselves (Hiraoka et al., 2009) and (b) that an agnosia for static forms does not extend to the same forms when in motion (Botez and Sebrãnescu, 1967), consistent with the suggestion that there may be a separate dynamic form system in the brain (Grossberg, 1991; Zeki, 1993). Our interest in mentioning the brain areas critical for form is (a) that regardless of whether the brain areas critical for face perception also respond to objects, other, distinct, cortical areas have been reported to be involved in object representation and, so far, these have not been implicated in face or body perception; (b) that the areas critical for face recognition should also be responsive to objects complicates the picture somewhat on the one hand while emphasizing a critical feature on the other, namely that the brain reaction to distorted faces and bodies is different from its reaction to distorted objects (see section Consequences of Violating the Essential Configuration of Faces).

### Inherited templates for facial and body recognition

Evidence that we are born with a capacity to recognize and register essential configurations that qualify stimuli as a face are present at birth or very soon (within hours) thereafter is shown by the fact that children react very early on—within a matter of hours—to faces, in that they orient more readily toward simple face-like patterns (Goren et al., 1975; Johnson et al., 1991). But what exactly they are reacting to is not universally agreed on. One view is that we are born with some kind of inherited “template” that approximates a face and another is that it has more to do with asymmetries in what appears in the upper and lower field of view, the reasoning being that new-borns prefer patterns in which more elements appear in the upper field of view (eyes) than in the lower (mouth) (Simion et al., 2002; Cassia et al., 2008). A third view may be that the intimate contact between infant and parent privileges the face through a rapid plastic process that facilitates the recognition of faces (Johnson, 2005). These arguments, though of substantial interest in the context of the neural determinants of facial perception, are of little interest for our present purposes because, whichever of the hypotheses turns out to be valid, the net result, perceptually, is that new-borns orient preferentially to faces or face-like stimuli, thus suggesting that there is something robust, or becomes rapidly robust, about configurations that are face-like. Whether due to an inherited concept (Zeki, 2008) for faces or face-like configurations or a privileged *plasticity* that favors the recognition of face-like stimuli, it is clear that there is a very early recognition of, and preference for, face-like stimuli. Hence, Bacon was subverting something very privileged in visual perception.

The perception of bodies has not been studied as extensively, but there are reasons to suppose that there are also essential configurations that qualify stimuli as being that of bodies. The evidence comes principally from electroencephalographic (EEG) recordings from the brains of 3–4 month old infants, who appear to be able to recognize bodies (de Gelder, 2006).

By contrast, there is no similar essential configuration to qualify an object, and where there is one through exposure and training, it can adapt rapidly to a new configuration that is radically different. One need only refer to the example of planes, from simple twin-engined turboprop planes, to drones, to jumbo jets, to variable swing-wing aircraft, to realize that there are many configurations that can fit the (acquired) concept of a plane (for before there were planes there was no acquired concept of them). Nor does there appear to be a distinct and privileged mechanism for early and rapid acquisition of a template for objects. Here it is interesting to note that, even in adult life, monkeys can be trained to learn new configurations of objects and discriminate them as a category even if they had not seen the particular example before (Logothetis et al., 1995). Whether rapidly acquired through a privileged plasticity or not, the templates for faces and bodies are not modifiable, in the sense that those for objects can be modified (see section Consequences of Violating the Essential Configuration of Faces).

### The holistic representation of face and body

**Figure 4:**
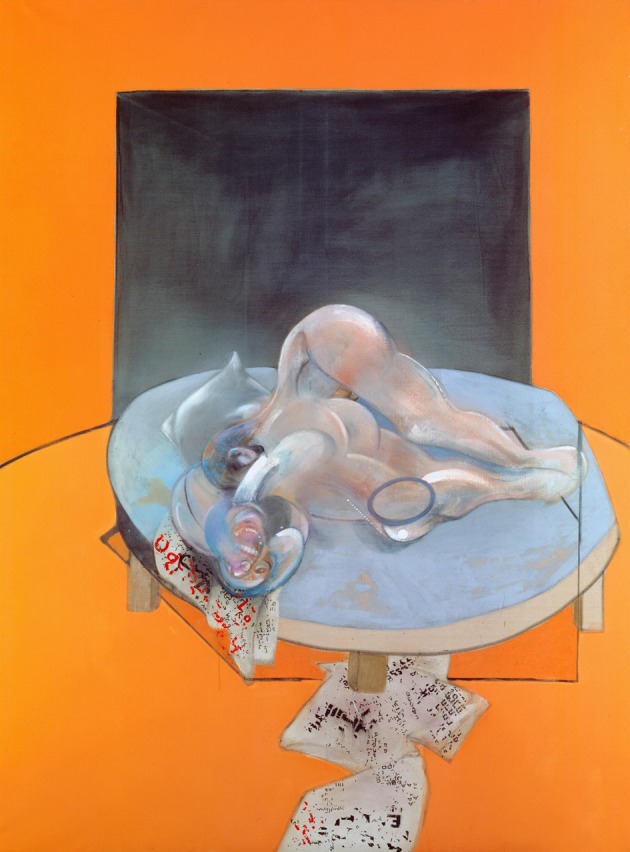
**Francis Bacon—*Triptych—Studies of the Human Body* 1979 (detail of center panel)**. © The Estate of Francis Bacon. All rights reserved. DACS 2013.

**Figure 5:**
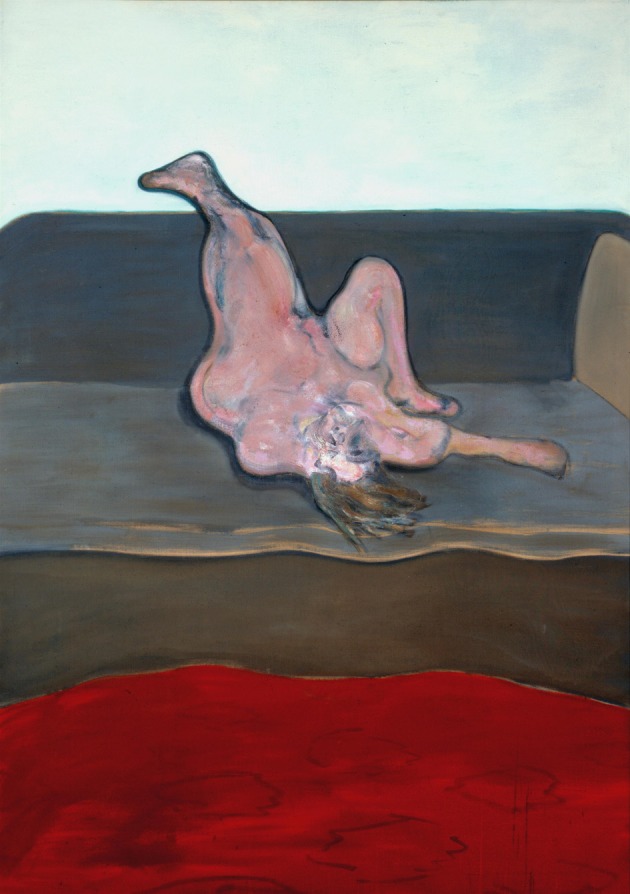
**Francis Bacon—*Reclining Woman*, 1961**. © The Estate of Francis Bacon. All rights reserved. DACS 2013.

While painting disfigured and mutilated bodies and faces, Bacon nevertheless maintained a generally holistic representation that makes it easy to discriminate his paintings as being of faces or bodies. It is commonly accepted that face representation is holistic. Evidence for this comes partly from studies of the so-called “inversion effect,” by which is meant the relative difficulty of recognizing faces when they are inverted, although Bacon himself rarely painted inverted faces and bodies, Figure 4 being a somewhat rare exception and Figure 5 (*Reclining Woman*, 1961) a more extreme version, in the total inversion and disfiguration of the human face and body. The inversion effect has been proposed as demonstrating the importance of configural, relational, information in facial recognition. It is not actually limited to faces, since objects in general become more difficult to recognize when inverted (Haxby et al., 1999); but inversion has a disproportionately large effect on facial recognition compared to the recognition of objects (de Gelder and Rouw, 2000). Many prosopagnosia studies also attest to the fact that the deficit is holistic, in the sense that it leads to an incapacity to recognize a face while sparing the ability to recognize its constituents, such as the eyes or the nose (Kimchi et al., 2012), that the whole is other than the sum of the parts, in Gestalt language. It is, in short, the relationship of the constituent parts that is critical, and constitutes the essential configuration. It is interesting to note here that a patient suffering from object agnosia but not prosopagnosia was capable of perceiving a face made up of objects (the Arcimboldo Effect), without being able to recognize what the constituent objects were (Moscovitch et al., 1997), implying that a given essential configuration or arrangement, no matter what the constituents that make up that configuration might be and no matter how distorted the constituents are, provided they bear the essential relationship to one another to constitute a face, are sufficient to qualify a face as a face.

The neural consequences of inversion are controversial, in line with the controversy as to whether there are “face modules” in the brain or whether there are extended brain regions in which objects are represented, of which faces constitute one category. There is general agreement that face inversion diminishes the response to faces in the FFA and the temporal face regions, and has a selective and dramatic effect on the responses to faces in regions which are responsive to houses (Haxby et al., 2000). This raises an interesting question: if knowledge of faces and objects are both acquired through expertise, as has been argued (Gauthier and Nelson, 2001 for a review), the larger perceptual susceptibility of faces and bodies to inversion implies that different mechanisms are at work, or perhaps that the neural mechanisms underlying one kind of representation are more labile than those underlying the other. Bacon appears to have opted instinctively for the less labile representation to deliver his visual shock.

Inversion of faces, as of bodies, also results in slower reaction times and higher error rates for identification (Reed et al., 2003) and it is inversion of the whole rather than of components that produces these results (see also the “Thatcher Illusion,” Thompson, 1980). Indeed, even distorted faces (ones in which the eyes are positioned asymmetrically) are processed holistically (de Heering et al., 2012). Crucially, inverted faces lead to a pattern of cortical activation that is distinct from that produced by upright faces and resembles more closely the activation pattern produced by viewing objects (Haxby et al., 1999), as if an inverted face becomes coded as yet another object. This implies again a difference in the neural mechanisms regulating the representation of the two. Inversion has a disproportionately large effect on the recognition of body postures (Reed et al., 2003). Distorted bodies also have a significant effect on brain-evoked potentials (Gliga and Dehaene-Lambertz, 2005), suggesting that the perception of bodies may also be facilitated by some inherited neural template, which may however also be facilitated through expertise.

**Figure 6:**
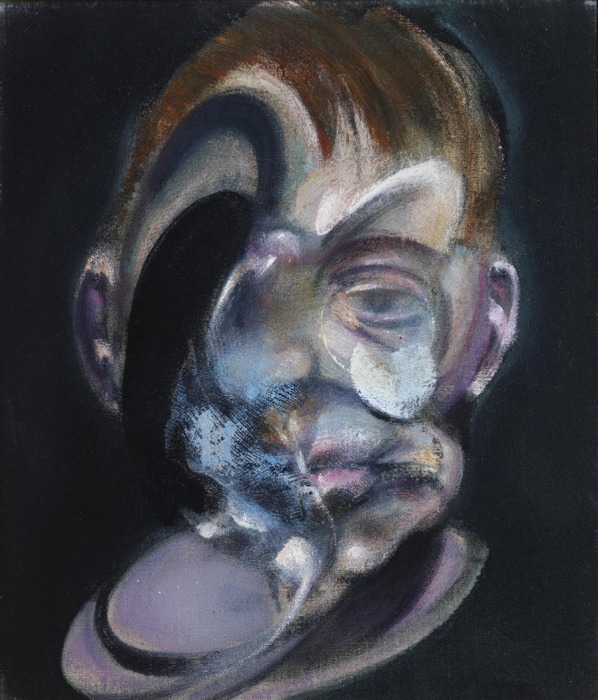
**Francis Bacon—*Self Portrait*, 1973**. © The Estate of Francis Bacon. All rights reserved. DACS 2013.

**Figure 7:**
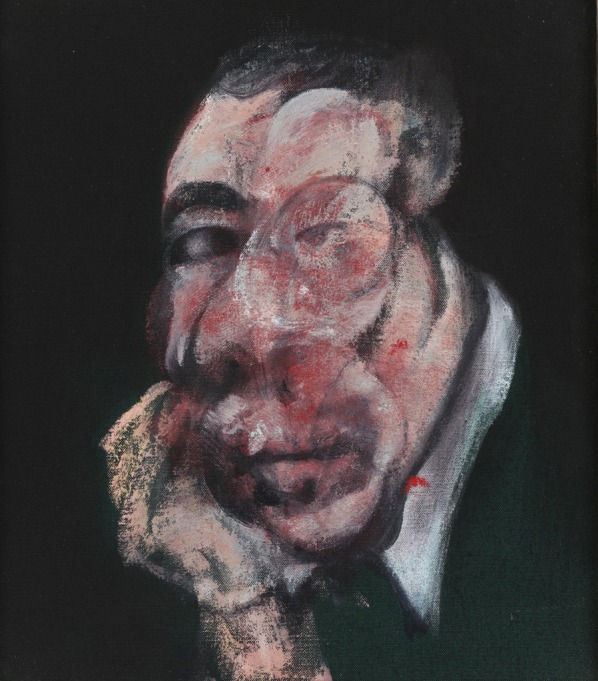
**Francis Bacon—*Head III*, 1961**. © The Estate of Francis Bacon. All rights reserved. DACS 2013.

The mutilation and disfiguration of faces and bodies in Bacon's work is largely restricted to the constituents but does not affect the relationship of these constituents to one another, hence maintaining their holistic aspect and allowing them to be recognized easily as faces or bodies. Only rarely is the relationship of the constituents altered, as in his *Self Portrait* (Figure 6), which violates somewhat the norms of a face in the absence of one eye, and the depiction of a severely distorted jaw with an abnormal relationship to mouth and nose. Otherwise, his distortions are of constituents which, though bearing a correct relationship to one another, may be unequal in size or severely asymmetric. The portrait in Figure 7 has an essential configuration that is recognizable instantly as a face, but it is a highly abnormal one, with one side being out of proportion with the other. Hence, in terms of our definition given above, the pictures contain not only the essential configuration necessary to result in activity—though apparently an abnormal one—in the areas critical for face perception, but in addition arouse strong negative emotions and also almost certainly entail activity in the amygdala and insula (see below section A Fast Route for the Recognition of Facial and Body Stimuli).

## The effect of distortions of face and body on cortical activity

The distortion of faces and bodies is more severe in some of Bacon's paintings than in others but very few can be said to render faces and bodies normally. Distortions in general, even those that are much less severe than the ones crafted by Bacon, lead to a pattern of cortical activity that is somewhat different from the one produced when humans view normal faces and bodies, although it should be emphasized that images of “distorted” bodies and faces used in the experiments described below were nowhere as extreme or as distorted as the ones depicted by Bacon in his paintings. In particular, the amplitudes of the responses evoked by viewing faces and bodies are reduced by viewing distorted versions of both (Gliga and Dehaene-Lambertz, 2005). It is, again, noteworthy that object inversion and distortion, which Bacon generally avoided, does not produce similar results (Boutsen et al., 2006).

**Figure 8:**
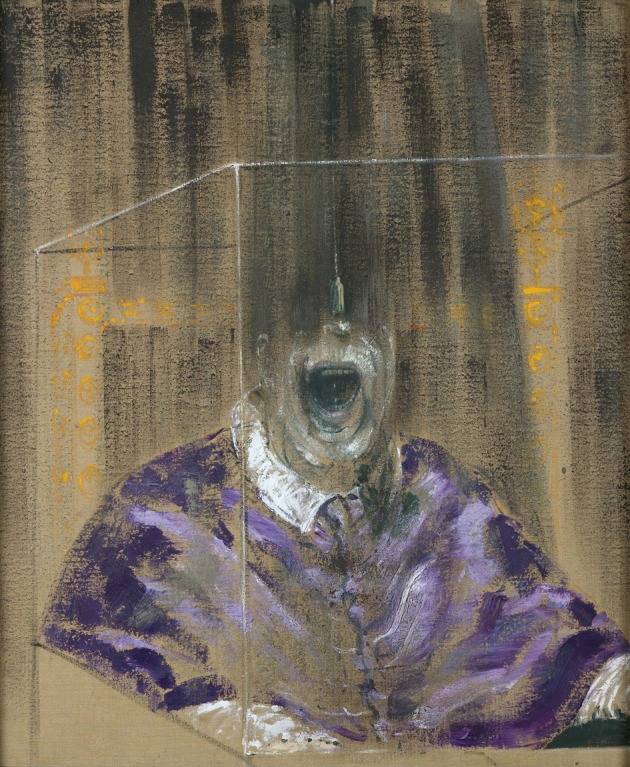
**Francis Bacon—*Head VI*, 1949**. © The Estate of Francis Bacon. All rights reserved. DACS 2013.

**Figure 9:**
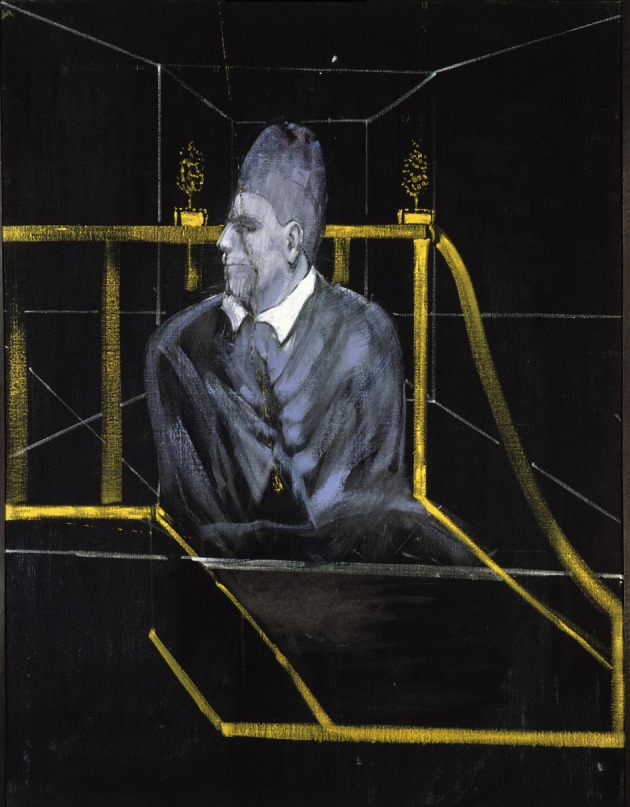
**Francis Bacon—Study for Portrait II, 1953**. © The Estate of Francis Bacon. All rights reserved. DACS 2013.

One of the most famous portraits of Bacon is inspired by Diego Velazquez's painting of Pope Innocent X, a painting which Bacon never really saw but worked from photographs of it alone. Bacon may have wanted to depict the human cage in which even someone so special, as he said, as the Pope is confined but the Pope is not the only figure to be so confined in Bacon's similar drawings. It has been suggested that the paintings are a reaction to his relationship with his father and that they were influenced by a scene from Eisenstein film *Battleship Potemkin* or by Nicholas Poussin's *The Massacre of the Innocents*, where a mother is crying in agony at the murder of her child, or perhaps both. Whatever their psychological and artistic origin, the Pope drawings nevertheless show an unaccustomed picture, of someone screaming, even if the face of the Pope is not as mangled as those in many of his other portraits. In *Head VI* (Figure 8), barely half the face of a screaming pope is visible, suggesting a profound abnormality characteristic of his other depictions of popes and cardinals. They thus also constitute a departure from a sort of distortion of what qualifies a face as a face. On the rare occasions when he portrayed, in similar conditions, a much more normally appearing face [Figure 9 (*Study for Portrait II*, 1952)], the impact is much less severe and the painting correspondingly much less arresting.

The list of distortions is hardly worth describing in detail; about the only general but accurate statement that can be made of all his paintings is that they are agonized, mutilated and savaged portraits. Cecil Beaton, the English photographer, recounts in his autobiography his shock at seeing Bacon's portrait of himself where, “The face was hardly recognizable as a face for it was disintegrating before your eyes, suffering from a severe case of elephantiasis; a swollen mass of raw meat and fatty tissues. The nose spreads in many directions like a polyp but sagged finally over one cheek. The mouth looked like a painful boil about to burst… ” (Peppiatt, 1996, p 226). Bacon himself preferred to work from photographs rather than have models in his studio, especially in his later years, “to avoid, as he said, inflicting on them in their presence the injury which he did to them in paint” (Peppiatt, 1996, p 204). Indeed, it is said that when Lucien Freud came to Bacon's studio to pose for a portrait, he found that it was almost finished, with Bacon insisting that he only needed to work on the feet!

It is interesting to note here that human-animal complexes—as in Egyptian art and in particular the sphinx—which Bacon greatly admired and which could be regarded as “distorted” representations of both humans and animals, are not nearly as unsettling or disturbing as the disfigured paintings of Bacon, either those of faces alone, or those of bodies, or of the two together. We suppose that this is because, although the two are combined in a departure from what humans usually experience, nevertheless the two neurally separately represented entities—bodies and faces—are normal and neither would constitute an “assault” on the nervous system. By contrast, when Bacon used the sphinx as a template for his paintings, both the body and the face were distorted (see Francis Bacon, *Oedipus and the Sphinx after Ingres*).

**Figure 10:**
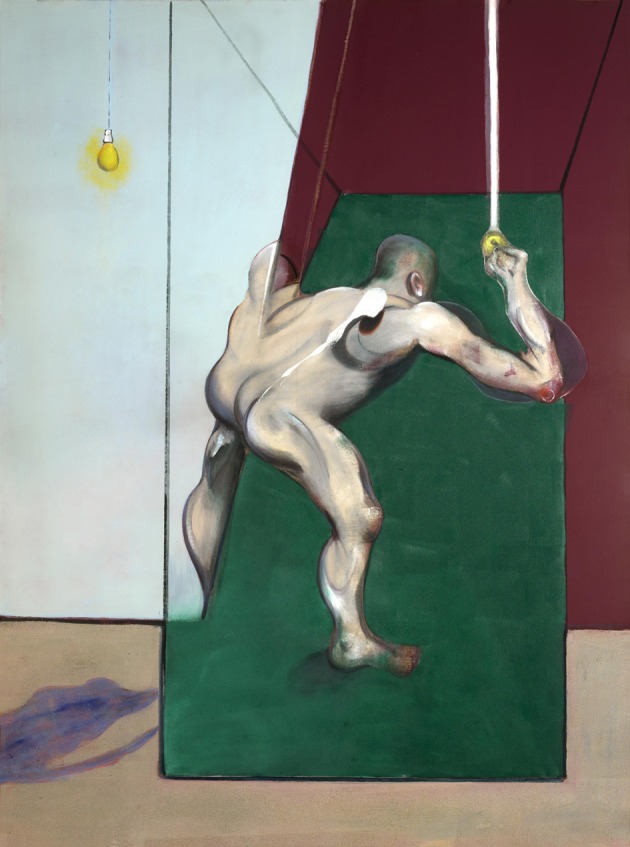
**Francis Bacon—*Study from the Human Body: Man Turning On the Light*, 1973**. © The Estate of Francis Bacon. All rights reserved. DACS 2013.

No less deformed in Bacon's paintings are the bodies; indeed few of his paintings, if any, can be said to escape that savage disfigurement. There is no particular part of the body that is privileged in this regard but what is interesting is that, even when a segment, for example the torso or the legs, is spared, the general impression gained by the viewer is a total disfigurement, suggesting a holistic representation of the body. His *Study for a Portrait* (1971) is a typical example of a mangled body, which has one or two “normal” features, in this case the foot, which nevertheless is in a somewhat abnormal position. *Study from the Human Body: Man Turning on the Light* (Reynolds, 2007) (Figure 10) has a more or less normal appearance in one half and a much distorted one in the other which, if bodies are processed configurally, would amount to distortion. Such examples may be multiplied, but it is interesting to note that, especially with his depictions of the human body, the ordinary objects incorporated into the paintings are virtually always undistorted.

The perceptual classification of a face or body as happy or threatening or sad or fearful also depends upon given specific configurations. It is common knowledge that upturned corners of the mouth are one element signifying a happy face while downturned ones signify the opposite. Here, another innovation in Bacon's works intrudes—his faces are neither happy nor sad, neither threatening nor comforting, neither fearful nor welcoming. Instead, they are all mutilated and usually savagely so; they are, in Peppiatt's words, “unusual” and “sinisterly unpleasant.” Hence, what Bacon has achieved is to trample over such configurations that allow the rapid classification of the emotional envelope on a face or a body into the above categories.

### A fast route for the recognition of facial and body stimuli

In his book, Peppiatt states that Bacon's intent was to produce work such “that the nerves are immediately alerted to *something unusual*, something sinisterly unpleasant, before the image has spelled itself out in the brain” (Peppiatt, 1996). Most of his paintings alert one to something unusual, even his relatively normal ones of the *Screaming Pope*. There is evidence that the emotionally disturbing rendering of faces and bodies engages a fast neural system, but whether this occurs before the image has “spelled itself out in the brain” is not certain. It is to be noted that objects can also be distorted but do not have nearly the same emotional impact as distorted faces and bodies and, moreover, that Bacon himself rarely distorted objects and when he did so, it was very mild and produces no emotional impact at all.

When the faces viewed have a “sinister” and therefore strong emotional component (both common in Bacon's paintings), there is activation of the amygdala (Morris et al., 1996; Hadjikhani and de Gelder, 2003; Sato et al., 2011) as well as of the insula (Krolak-Salmon et al., 2003), although neither has been shown to be engaged when neutral faces are viewed. It has been suggested that viewing a fearful face leads to fast, short-latency activation (at about 100 ms after exposure) of the amygdala before spreading to the cortex (Krolak-Salmon et al., 2004). More recent evidence shows that the latency of response from the sub-cortical centers involved is not very different from latencies in areas such as the OFA when subjects view neutral faces. Fearful faces activate the amygdala rapidly (in the 50–150 ms time frame), while a transcranial magnetic stimulation study suggests the earliest activity in the OFA occurs at 60–100 ms for neutral faces (Pitcher et al., 2007), with a later component at 150 ms (Hung et al., 2010).

The facial recognition route which registers rapidly extreme expressions on a face or a body such as fear or disgust, is more “primitive” in the sense that it is activated by low spatial frequencies (coarse visual information) and is independent of the precise identity of the person viewed (Vuilleumier et al., 2003; Maratos et al., 2009). The sub-cortical routes seemingly influence strongly face perception but can act autonomously, since subjects can recognize the valence on a face when faces are viewed without conscious awareness of the face itself (de Gelder et al., 2005), even if the sub-cortical route relays signals to the corresponding cortical zones and modulates activity in them (Johnson, 2005). This suggests that the emotional component—fear, disgust, (as is so common when viewing Bacon's paintings)-is recorded as rapidly as the face itself. Hence, the sub-cortical system may be instrumental in alerting the brain, with very brief latencies, that a stimulus recognized as a face has something unusual about it.

It is likely that the sub-cortical system is used in the demonstrated newborn preference for faces (Johnson, 2005). This route may in fact not only modulate cortical responses but also be indicative of a system involved with facial recognition that acts in parallel with the high frequency system, which identifies details on the face as well as facial identity. Thus, while the recognition of a stimulus as containing the “primitives” of a face might depend upon a sub-cortical system and on low spatial frequencies, the process appears to become more “corticalized” as refinements due to experience are added and recognition is not only of a face as such but the identity of the face (Johnson, 2005).

To our knowledge no parallel studies have been performed to learn whether there is a sub-cortical or cortical system that reacts to bodies presented in low spatial frequencies. Nor has any fast, sub-cortical route for object recognition been reported.

### Unconscious emotional impact of disfigured bodies and faces

Bacon often emphasized that his work came from the “unconscious.” “I've made images that the intellect can never make,” he told Melvyn Bragg emphatically (Bacon, interviewed by Bragg, 1985). He also often stated that he produced some of his most prized works, such as *Three Figures at the Base of a Crucifixion* (1944) (Tate, 2013a) [of which there is also a second version (Tate, 2013b)], when in an inebriated state and not capable of clear thinking, thus perhaps emphasizing the predominance of what he supposed is the “unconscious” element. Bacon reputedly was inspired by a number of sources for this painting, including Greek mythology as well as the work of Pablo Picasso. Taken together with his avowed aim of attacking the nervous system before things get spelled out in the brain, he is perhaps emphasizing that his paintings are originating from the “unconscious” and are destined for the “unconscious.” Of course, what Bacon means by the “unconscious” is never spelled out clearly or defined. The meaning we would like to attach to it is more specific; we mean by it a severe mutilation and distortion of what constitutes a normal face that is registered in the brain even when the subject is not consciously aware of having viewed such a face. Violations of essential configurations are experienced consciously and have, as a consequence, an emotional dimension that is also experienced consciously. But there appears to be also an unconscious dimension that mediates the experience; subjects can discriminate the emotional valence on a face even when not consciously aware of the face, especially if the expression is fearful (Bertini et al., 2013). Here it is important to notice, once again, that the “fearful” faces used in such experiments are not nearly as unusual as those depicted by Bacon. The rapid activation of amygdala and insula by emotional stimuli which can be registered “unconsciously,” implies that, for the ordinary viewer, a Bacon painting is registered through the two parallel systems, cortical and sub-cortical, with a dominant sub-cortical emotional registration occurring through structures such as the amygdala and insula. It is hard to escape the conclusion that the sub-cortical system is the emotionally more dominant one, since it is capable of responding even in the absence of an acknowledged “awareness” of the stimulus. The adjectives used to describe Bacon's work—“repellent,” “mutilated,” “hell”–serve to describe well the strong emotional component in his work, a component which seemingly would activate the emotional branch of the face-recognition system powerfully. Disregarding the religious connotation in the title of *Three Studies for Figures at the Base of a Crucifixion*, it is evidently a painting of some horrifically deformed animal(s), so deformed that it is hard to tell the species or indeed whether it is an animal at all. Yet, we emphasize again, there is nothing extraordinary about the geometric configurations against which the animals are set. Especially in the second version of the *Three Studies*, the geometric lines are normal and the tables are easily recognizable as tables though the central one could easily be conceived of as the somewhat bizarre creation of a modern artist.

**Figure 11:**
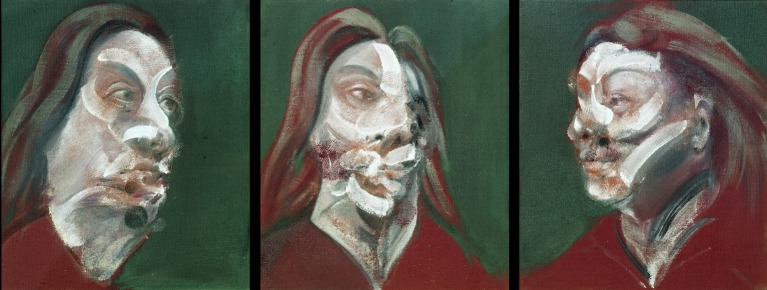
**Francis Bacon—*Three Studies of Isabel Rawsthorne*, 1965**. © The Estate of Francis Bacon. All rights reserved. DACS 2013.

It is to be noted, however, that the emotional valence on some of his portraits or bodies are hard to classify as fearful or shocking or threatening; they are departures even from the norms that we associate with such emotions. How, for example, is one to categorize, in terms of emotions, the triptych portrait of Isabel Rawsthorne, whom Bacon considered to be “a very beautiful woman” (Bragg, 1985), shown in Figure 11? Severely mutilated may be a more appropriate term, especially for the central portrait; what is not in doubt is that all three represent significant departures from normal faces and normal emotions, be they emotions of fear or happiness. To that extent they are subversions of the brain's normal, expected, experience of faces and hence constitute and represent a threat. It would be interesting to learn how such distortions, which can be qualified only as unusual but not necessarily as ugly or threatening, affect the pattern of activity in both the cortical and sub-cortical systems that are important for facial recognition.

## Human artefacts in bacon's paintings

**Figure 12:**
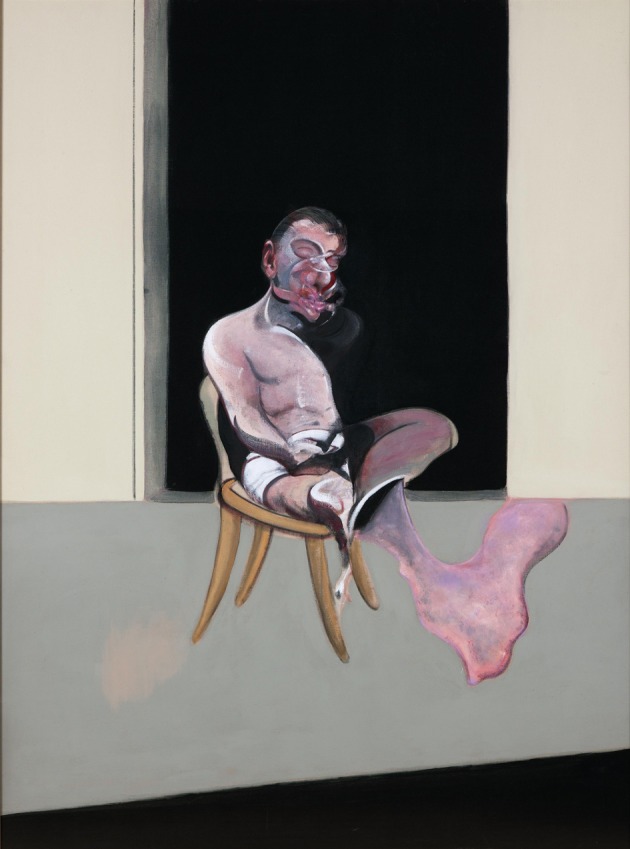
**Francis Bacon—*Triptych August* 1972 (right hand panel)**. © The Estate of Francis Bacon. All rights reserved. DACS 2013.

**Figure 13:**
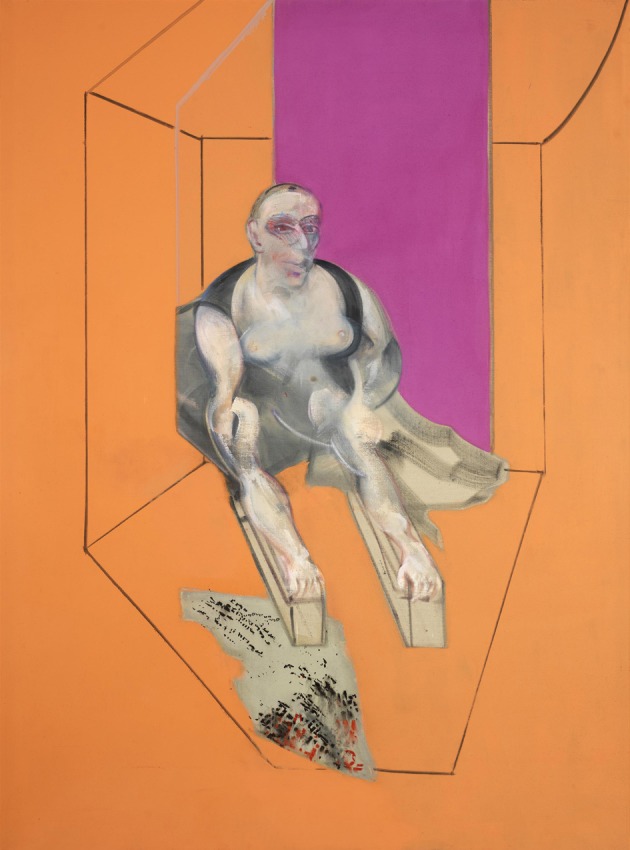
**Francis Bacon—*Sphinx—Portrait of Muriel Belcher*, 1979**. © The Estate of Francis Bacon. All rights reserved. DACS 2013.

**Figure 14:**
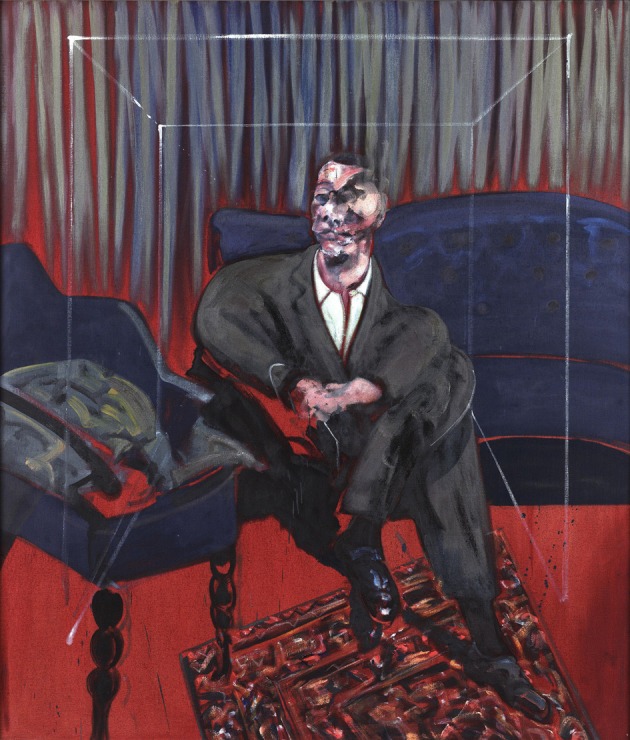
**Francis Bacon—*Seated Figure*, 1961**. © The Estate of Francis Bacon. All rights reserved. DACS 2013.

We have alluded repeatedly above to the difference in Bacon's paintings between faces and bodies on the one hand and objects on the other, the former being severely distorted and mutilated while the latter escaped such violence from one who thought that the whole of life is violent. We give a few more examples below, to emphasize the point: The chair on which the man of Figure 12 sits is fairly normal as is the window or door behind. Equally, there is nothing unusual in the lines that constitute a sort of cage in which the person portrayed in Figure 14 sits. Bacon claimed that he used these lines only as a kind of frame for what he was painting. In Figure 13, the cage could be in a bi-stable state and somewhat unusual in shape but other than that there is nothing about it that is shocking, even in spite of its somewhat unusual shape. Equally, the furnishings of Figure 14 are all fairly normal, while the face of the sitter is severely deformed. Such examples may be multiplied and attest to one difference between his rendering of bodies and faces on the one hand and objects on the other: he deformed and mutilated the former but left the latter largely intact.

## Consequences of violating the essential configuration of faces

Superficially, any unusual visual input may be considered to be a visual shock but most of these are momentary and quickly adapted to. A very unusual artifact, one which departs from the general class of artifacts to which it belongs (say of planes or cars), may at first sight constitute a visual shock in the sense that it is an unaccustomed departure from the norm. With repeated viewing and time, however, it ceases to be a shock but comes to be accepted as commonplace; but this does not seem to be true of visual stimuli for which we have an ingrained or possibly inherited predisposition (Chen and Zeki, 2011).

In further evidence of the robustness of the neural templates—whether inherited or rapidly acquired after birth—for essential configurations that qualify a visual stimulus as a face, are experiments inspired by Bacon's work, which have aimed to chart the differences that underlie the perception of violated faces and violated human artifacts such as cars or planes. Violated faces, unlike normal faces and violated human artifacts, result in activation of dorsolateral prefrontal cortex (DLPFC) and parietal cortex. This activation is resistant to prolonged viewing of violated faces (up to one month), in that viewing abnormal faces for that period does not decrease activity in that cortex but actually enhances it. This is interesting because the DLPFC gives a strong reaction to unpredictable stimuli or to departures from what is considered normal. For example, although the DLPFC does not appear to be active when objects are dressed in colors with which they are normally associated, it is active when humans view objects dressed in un-natural colors, that is to say colors with which they are not usually associated (Zeki and Marini, 1998). The strength of activity in the DLPFC appears to decrease with prolonged exposure to such unpredictable stimuli (Raichle et al., 1994; Rainer and Miller, 2000; Fletcher et al., 2001). That the activity in the DLPFC should have increased when viewing violated faces even after prolonged exposure to such stimuli implies (a) that we do not adapt easily to the *concept* of violated faces and (b) that the significant configuration that qualifies a stimulus as a face is much more robust than the configurations that characterize the recognition of artifacts acquired through experience, and hence any departures from it are strongly registered. It is interesting to note in passing that violation of spatial relations (which Bacon did not indulge in) are also resistant to adaptation over a similar period (Chen and Zeki, 2011).

Whether the brain has specialized “face modules” or whether faces constitute one category processed in a large cortical zone which also processes other categories, that violation of faces should lead to strong and enduring activity within parietal cortex and the DLPFC while violations of human artifacts should not, leads naturally to the supposition that the neural mechanisms regulating the two categories (and probably bodies as well) differ significantly, although what this difference is must remain conjectural for the present.

What we are suggesting is that Bacon, unknowingly, used a robust system based on an inherited concept and violated it to produce his shock. That we do not become readily adapted to such violations, although we become adapted to violations of human artifacts, perhaps accounts for the enduring shock effect that Bacon's work, almost all of which violates faces and bodies, has.

There are of course many other aspects of Bacon's work that we could discuss, but this would enter too much into a world of speculation. While it is clear that different categories of animals elicit a reaction from the visual brain, the effect of deformation of animal faces and bodies on brain activity has not been studied in any detail. But it is probably safe to assume that deformation of animals has a similar effect—though possibly a less pronounced one—than deformation of human faces and bodies. Bacon commonly painted animals and in some of his paintings he combined a human body with an animal face, or vice versa, or incorporated some elements of an animal into the depiction of a human.

## Conclusion

What then are the insights of neurobiological and neuroesthetics interest that Bacon's paintings provide, as material for future experiments?

We have based much of our argument on essential configurations that allow us to classify a stimulus as that of a face or a body, a theoretical construct that may yet lead to important experiments and insights. We have used previous results to show that distortions of that essential configuration results in a pattern of activation that is consistently different from the one obtained when viewing configurations that satisfy the template of what constitutes face or a body. We have argued that such departures can have consequences. One of these, which Bacon exploited, is that viewing configurations that depart from the essential configurations has, as a correlate, a strong activation of sub-cortical structures such as the amygdala and the insula, an effect that can be produced even when subjects are “unaware” of the stimulus; moreover, departures are resistant to adaptation, in that continual exposure does not diminish the response obtained from the DLPFC and parietal cortex, as repeated exposure to unusual human artifacts apparently does.

This raises a host of interesting questions. The first among them is related to the representation of faces, bodies and objects in the brain. Whether they are represented in discrete groupings within a larger cortical area or whether each of these categories is separately represented, Bacon's paintings raise the question of a separate and privileged access to the brain's emotional systems from the representation of faces and bodies compared to ordinary man-made objects. If so, it is likely that groupings or modules representing faces and bodies have different connections with the brain's emotional system, through routes that remain to be determined. Equally interesting in this context is that the representation of faces and bodies appears to be much more robust, which implies that there is less room for experience to modify that representation in the way that representation of human artifacts can be modified, a suggestion supported by the experiments of Chen and Zeki (2011). This implies that the connections of the latter are much more plastic than those of the former, making it interesting to uncover the different mechanisms that regulate plasticity in these different representations. This is also likely to be reflected in the mechanisms regulating the formation of concepts for different attributes. The enduring shock element in Bacon's paintings, even after repeated viewing, speaks in favor of a pronounced resistance to modifying the concept of a face or a body; by contrast, concepts of human artifacts are much more modifiable and less resistant to change. Hence, it follows that the determinants of concept formation are much less plastic for faces and bodies, the brain apparently not tolerating departures from a primitive significant configuration for them.

Next comes the question of routing of visual signals to and from a given area of the brain. It is important to realize that faces and bodies, whether ugly, neutral or beautiful, are processed through common structures—the OFA, the FFA and other areas detailed above. At some point in these pathways, a neural decision must be taken to forward the results of the processing to one part of the emotional brain or another. This raises the question “at what level, in the face and body processing pathways, is the routing of signals to one of the destinations made?” a question that applies equally to beautiful and ugly faces. It is also interesting to learn when and how signals are not routed to the emotional centers or routed to them without eliciting a strong and detectable response, as happens with neutral faces. This of course amounts to a neurobiological question of general interest, for all cortical areas have multiple inputs and outputs and whether all the outputs from an area are active when the area undertakes an operation or whether they are active only when the area undertakes a particular operation is an important question to address (Zeki, 1993). In our context, this can be more precisely formulated by asking whether departures in significant configuration in one direction activate certain outputs from the area while departures in the other direction activate other pathways.

This also raises the question of what constitutes, in terms of responses from a given area, say the FFA, a departure from an essential configuration, i.e., does it lead to an increase or decrease in firing of cells in the area or does it lead to a different pattern of active cells. In theory at least, it should be possible to study this by using imaging techniques that can determine whether the pattern of activity in a given area differs according to departures from the essential configuration.

Hence, Bacon's work raises a host of interesting and important problems, not only in the somewhat specific domain of the neural mechanisms regulating face and body perception but the more general neurobiological problem of what it is that determines the routing of signals to one destination or another, given that each area has multiple outputs.

### Conflict of interest statement

The authors declare that the research was conducted in the absence of any commercial or financial relationships that could be construed as a potential conflict of interest.
